# CBF-1 Promotes the Establishment and Maintenance of HIV Latency by Recruiting Polycomb Repressive Complexes, PRC1 and PRC2, at HIV LTR

**DOI:** 10.3390/v12091040

**Published:** 2020-09-18

**Authors:** Adhikarimayum Lakhikumar Sharma, Joseph Hokello, Shilpa Sonti, Sonia Zicari, Lin Sun, Aseel Alqatawni, Michael Bukrinsky, Gary Simon, Ashok Chauhan, Rene Daniel, Mudit Tyagi

**Affiliations:** 1 Center for Translational Medicine, Thomas Jefferson University, 1020 Locust Street, Philadelphia, PA 19107, USA; LakhikumarSharma.Adhikarimayum@jefferson.edu (A.L.S.); shilpasonti@gmail.com (S.S.); Aseel.Alqatawni@jefferson.edu (A.A.); Rene.Daniel@jefferson.edu (R.D.); 2 Department of Basic Science, Faculty of Science and Technology, Kampala International University-Western Campus, P.O. Box 71, Bushenyi, Uganda; hokello.joseph@kiu.ac.ug; 3 Section of Intercellular Interactions, Eunice Kennedy-Shriver National Institute of Child Health and Human Development, Bethesda, MD 20892, USA; sonia.zicari@opbg.net; 4 Department of Pediatric Medicine, The Bambino Gesù Children’s Hospital, 00165 Rome, Italy; 5 Division of Infectious Diseases, Department of Medicine, George Washington University, Washington, DC 20037, USA; lsundec@gmail.com (L.S.); gsimon@mfa.gwu.edu (G.S.); 6 Department of Microbiology, Immunology and Tropical Medicine, George Washington University, Washington, DC 20037, USA; mbukrins@gwu.edu; 7 Department of Pathology, Microbiology and Immunology, University of South Carolina School of Medicine, Columbia, SC 29209, USA; acniwar@gmail.com

**Keywords:** HIV-1, latency, epigenetics, CBF-1, PRC1, PRC2, chromatin, transcription

## Abstract

The C-promoter binding factor-1 (CBF-1) is a potent and specific inhibitor of the human immunodeficiency virus (HIV)-1 LTR promoter. Here, we demonstrate that the knockdown of endogenous CBF-1 in latently infected primary CD4+ T cells, using specific small hairpin RNAs (shRNA), resulted in the reactivation of latent HIV proviruses. Chromatin immunoprecipitation (ChIP) assays using latently infected primary T cells and Jurkat T-cell lines demonstrated that CBF-1 induces the establishment and maintenance of HIV latency by recruiting polycomb group (PcG/PRC) corepressor complexes or polycomb repressive complexes 1 and 2 (PRC1 and PRC2). Knockdown of CBF-1 resulted in the dissociation of PRCs corepressor complexes enhancing the recruitment of RNA polymerase II (RNAP II) at HIV LTR. Knockdown of certain components of PRC1 and PRC2 also led to the reactivation of latent proviruses. Similarly, the treatment of latently infected primary CD4+ T cells with the PRC2/EZH2 inhibitor, 3-deazaneplanocin A (DZNep), led to their reactivation.

## 1. Introduction

The anti-human immunodeficiency virus (HIV) therapy, ART, has been highly successful in controlling human immunodeficiency virus (HIV) replication and maintaining the level of circulating HIV below the limit of detection. However, interruption of ART, even after decades of successful anti-HIV therapy, results in rapid and robust viral rebound [1,2,3]. The failure of ART to eradicate HIV is due to the creation of stable reservoirs of latently infected cells harboring slowly or non-replicating viruses. The majority of latent proviruses reside in resting memory CD4+ T cells, which provide a stable pool of latently infected cells with half-life roughly around 44 months [4,5,6]. These latent reservoirs are frequently replenished due to both the homeostatic proliferation of latently infected cells and the ectopic reactivation of latent proviruses followed by new rounds of infection, presumably owing to locally sub-optimal ART concentrations [7,8,9,10]. It is now well established that ART alone cannot eradicate latently infected cells, since the intensification of ART was also found to be ineffective in reducing latent reservoir [8,11]. Hence, developing therapeutic interventions with a focus on HIV eradication will require the precise definition of the molecular mechanisms responsible for both the establishment and maintenance of HIV latency, in order to either reactivate latent proviruses, so that they can be destroyed, or fossilize them forever like human endogenous retroviruses (HERVs).

As a retrovirus, the HIV replication depends on efficient transcription. HIV transcription primarily relies on the availability of the host cell transcription machinery, along with HIV’s own master transactivator protein Tat. Inefficient proviral transcription appear to be the major factor contributing to HIV latency. Numerous factors and multiple mechanisms are known to impair HIV transcription, and thus shown to promote HIV latency [9,12,13,14]. Notably, the type of epigenetic modifications and the resultant state of chromatin structures at the integrated HIV provirus provides critical signals that regulate transcription during both productive and latent HIV infections [9,15,16,17]. The role of repressive epigenetic modifications in supporting HIV latency is quite evident by the fact that their removal or inhibition leads to the reactivation of latent proviruses [15,18,19].

We previously described the important role of C-promoter binding factor-1 (CBF-1), a CSL (CBF-1, SuH and Lag-1) type transcription factor, in restricting HIV transcription during HIV latency [20]. CBF-1 is a key effector of Notch signaling pathways, which play a critical role in several developmental processes [21]. CBF-1 restricts the expression of several cellular genes that carry appropriate DNA-binding sites for CBF-1 by recruiting histone deacetylases (HDACs) containing corepressor complexes [20,22,23]. By performing experiments in both transformed and primary CD4+ T cells, we have established the role of CBF-1 as a potent repressor of HIV transcription [20,24]. We have demonstrated that CBF-1, after binding to specific sites in HIV LTR, recruits corepressor complexes containing HDACs (HDAC1 and HDAC3). HDACs subsequently deacetylate the core histones and facilitate the establishment of transcriptionally repressive heterochromatin structures at HIV LTR. The closed/compact heterochromatin structures restrict the flow of transcriptional machinery at LTR promoter and thus hamper HIV transcription and promote HIV latency [20,24].

The recent literature suggests that CBF-1 restricts cellular gene expression, not only through histone deacetylation, but also by inducing numerous other repressive epigenetic modifications, including the trimethylation of histone H3 at positions lysine 9 (H3K9me3) or lysine 27 (H3K27me3) [25,26,27]. The presence of H3K9me3 and H3K27me3 at LTR and their role in establishing heterochromatin during HIV latency have already been demonstrated, both by us and others [20,28,29,30,31]. In addition, we have also validated their physiological significance by showing the role of these repressive epigenetic modifications in establishing HIV latency in primary CD4+ T cells [24]. The formation of H3K9me3 is primarily catalyzed by two methytransferases, namely SUV39H1 and G9A [32]. The methylation of histone H3 at position 27 (H3K27me3) is mainly catalyzed by EZH2 and occasionally by EZH1 [33,34,35,36]. EZH2 and EZH1 are the main catalytic components of the PRC2 complex [37,38]. SUV39H1 and G9A frequently interact with different components of PRC1 complex [39,40]. PRC1 complex primarily inhibits transcriptional elongation via the monoubiquitination of histone H2A, but it is also involved in several other epigenetic transactions along with the PRC2 complex via different interactions among their subunits [38,41,42]. PRCs play an important role in both inducing and maintaining the silencing of several cellular genes. PRCs restrict cellular gene expression by simultaneously inducing various types of repressive epigenetic modifications involving both histones and DNA, as PRCs carry multiple chromatin modifying enzymes [38,43,44,45,46,47]. Consequently, PRCs-mediated epigenetic modifications regulate not only the transient gene silencing, but also the long-term silencing of the genes, such as of Hox genes and X-chromosome inactivation [48,49,50,51].

In this study, we show that CBF-1 is the factor that promotes the recruitment of PRCs at HIV LTR. Recently, the role of PRCs during both the establishment and maintenance phases of HIV latency was confirmed, and the presence of H3K27me3 at HIV LTR was documented [52,53,54,55,56]. We further established the physiological significance by showing the role of H3K27me3 during HIV latency in primary CD4+ T cells [24]. However, the identity of the factor that recruits PRCs at HIV LTR was obscure. Remarkably, most of the enzymes that catalyze epigenetic modifications are not able to bind directly to the DNA, and thus need to be recruited to DNA templates by various DNA binding proteins. Proteins such as CBF-1, YY1/LSF1, P50 homodimer, AP4, CTIP2, and thyroid hormone receptor recruit chromatin modifying enzymes in the form of multiprotein corepressor complexes to HIV LTR [20,30,57,58,59,60]. Using latently infected primary CD4+ T cells, we found that CBF-1 is the protein that recruits both PRC1 and PRC2 at HIV LTR. We confirmed that, by recruiting PRCs at HIV LTR, CBF-1 supports both the establishment and maintenance of HIV latency. Furthermore, we validated the direct role of PRCs in HIV latency, as their knockdown results in the reactivation of latent HIV.

## 2. Materials and Methods

### 2.1. Cell Culture, Cell Lines, Antibodies and Chemicals

The CD4+ T cells were isolated, either from tonsils obtained from routine tonsillectomy or from peripheral blood mononuclear cells (PBMCs) of healthy donors, using Ficoll–Hypaque (GE Healthcare, Chicago, IL, USA) gradient centrifugation. CD4+ T cells were purified by negative selection method using a MACS kit (Miltenyi Biotechnology, Auburn, CA, USA). CD4+ primary T cells and H80 cells were maintained in RPMI 1640 medium, supplemented with 10% fetal calf serum and 25 mM HEPES. CD4+ primary T cells were supplemented with recombinant human IL-2 (20 U/mL) (R&D Systems, Inc., Minneapolis, MN, USA). 293T cells were grown in Dulbecco’s modified Eagle’s medium (DMEM) supplemented with 10% fetal bovine serum. T-cell lines CEM and Jurkat were maintained in RPMI 1640 medium supplemented with 10% fetal bovine serum (FBS), penicillin (100 IU/mL), and streptomycin (100 μg/mL). All cells were grown at 37 °C, and in the presence of 5% CO_2_. Several of the antibodies were purchased from Santa Cruz, including anti-RNA polymerase II (RNAP II) (N-20 sc-899; F-12 sc-55492; A-10 sc- 17798), CBF-1 (Millipore, Burlington, MA, USA, AB5790; E-7 sc-271128; Sigma, Louis, MO, USA, AB384), CIR (C-19; H-1, sc-514120), mSIN3A (AK-11, G-11 sc-5299), HDAC-1 (H-51 sc-7872; H11 sc-8410, 10E2 sc-81598), HDAC-3 (H-99 sc-11417; A-3 sc-376957), p300 (C-20 sc-585; F-4 sc-48343), HP1α (C15 sc-10210; Active Motif 2HP-1H5).GAPDH (6C5 sc-32233; 0411 sc-47724), anti-β-actin (C-4 sc-47778), Spt5 (D-3 sc-133217), EED (H-300 sc-28701; Active Motif 41D) and p65 (F-6 sc-8008); Preimmune Rabbit IgG control (Cell Signaling, Danvers, MA, USA, #2729S), anti-phospho-Ser2 RNAP II (Active Motif 3E10; Abcam ab5095), acetyl-histone H3 (Upstate 07-593); SUZ12 (Cell Signaling, 3737S); EZH2 (Cell Signaling 5246S; Millipore 17-662); JARID1A (Abcam, ab65769); H3K9me3 (Upstate 07-442, Abcam ab8898-100); H3K27me3 (07-449; Upstate); RING1B (Active Motif 39663); BMI1(Active Motif AF27, Upstate F6). We also procured TNF-α (R&D systems), 3-deazaneplanocin A (DZNep, Cayman), α-CD3/CD28 antibodies conjugated to magnetic beads (Dynal Biotech, Oslo, Norway), and IL-2 (R&D Systems, Inc.).

### 2.2. HIV Lentiviral Vectors and Generation of VSV-G-Pseudotyped Viral Particles

The HIV-1-based lentiviral vectors pHR′P-PNL-mCherry and pHR′P-PNL-d2EGFP were constructed with either wild-type Tat or defective H13L Tat as described previously [20]. The construction of pHR′-PNL-Luc has also been previously detailed [20]. The small hairpin RNAs (shRNA) vector pHR′P-SIN-CMV-GFP was generated by cloning the CMV-GFP insert into the SacII to XhoI sites of the pHR′P-SIN-18 vector. The short hairpin RNA (shRNA) inserts were initially cloned into the pSuper vector (Oligoengine). The shRNA plus the H1 promoter were then cloned into pHR′P-SIN-CMV-GFP between the BamH1 and SalI sites, as detailed earlier [20,61]. The HIV-based lentiviral vector particles pseudotyped with the vesicular stomatitis virus glycoprotein (VSV-G) were produced using a three-plasmid co-transfection procedure [62,63]. The viruses were concentrated by ultracentrifugation, aliquoted and frozen at −80 °C until use.

### 2.3. Tyagi-Sahu Model to Generate Latently Infected Primary CD4+ T Cells

The latently infected primary CD4+ T cells were generated using our well established Tyagi–Sahu model system [24,64]. Briefly, the purified CD4+ T cells (>98% pure) from either PBMCs or tonsils were stimulated for 4 days with 25 μL of α-CD3/-CD28 antibodies conjugated to magnetic beads (Dynal Biotech) per million cells, along with 20 U/mL of IL-2. One million cells were infected with one of the VSV-G-pseudotyped HIV viruses expressing fluorescent protein gene through HIV LTR promoter. After 2 to 4 days, the fluorescent cells were purified by fluorescence-activated cell sorting (FACS). The pure population of infected cells was again amplified in the presence of α-CD3/-CD28 antibody-conjugated Dynal beads (25 μL/10^6^ cells) and 20 U/mL of IL-2 for 2 to 3 weeks. Fresh medium was added every 4 days to maintain a density of 1.5 × 10^6^ to 2.0 × 10^6^ cells/mL. Once it became 0.5 × 10^8^ to 1 × 10^8^, the cells were placed on 30% to 40% confluent H80 adherent cell mono-layer [24,64]. Every 2 to 3 days, half of the culture medium was replaced by fresh IL-2-containing medium, and every 2 weeks, the T lymphocytes were transferred to the fresh flasks of H80 feeder cells. Once most of the cells (>95%) lost GFP, we reactivated a fraction of cells via T cell receptor (TCR) stimulation by treating them with α-CD3/-CD28 antibody-conjugated Dynal beads (25 μL/10^6^ cells) and 20 U/mL of IL-2. Once we confirmed that most of the cells carry transcriptionally silent (latent HIV), we characterized the cells as detailed previously [24] and (Supplementary Figure S1). Those latently infected primary CD4+ T cells were then utilized in assays, before and after reactivating the latent provirus via TCR stimulation [24,64].

### 2.4. ChIP Assays and q-PCR

Chromatin immunoprecipitation (ChIP) assays were done following a previously described protocol [20,24]. To activate cells, we used either 10 ng/mL TNF-α (for cell lines) or 25 μL per 10^6^ cells of α-CD3/CD28 antibodies bound Dynal beads, along with 20 U/mL of IL-2 (for primary T cells). The chromatin was immunoprecipitated using different antibodies detailed in the antibodies section. Each sample (5%) was analyzed by quantitative real-time PCR (qPCR) to assess the amount of sample immunoprecipitated by an individual antibody. SYBR green PCR master mix (12.5 μL/sample; Bio-Rad, Hercules, CA, USA) combined with 1 μL of each primer, 5 μL of ChIPed DNA and water to a final volume 25 μL was analyzed by real-time q-PCR. The primers used were the following (numbered with respect to the transcription start site): promoter region of HIV-1 LTR (promoter) forward,−116, AGCTTGCTACAAGGGACTTTCC and reverse +4, ACCCAGTACAGGCAAAAAGCAG; nucleosome-1 region HIV-1 LTR (Nuc-1) forward +30, CTGGGAGCTCTCTGGCTAACTA and reverse +134, TTACCAGAGTCACACAACAGACG; glyceraldehyde 3-phosphate dehydrogenase (GAPDH) promoter forward, −125, CACGTAGCTCAGGCCTCAAGAC and reverse, −10, AGGCTGCGGGCTCAATTTATAG; GAPDH was also assessed by forward, −145, TACTAGCGGTTTTACGGGCG and reverse, +21 TCGAACAGGAGGAGCAGAGAGCGA.

### 2.5. Western Blotting

Western blotting was performed according to standard protocols. Briefly, nuclear extracts were run on acrylamide Tris-HCl buffered SDS-PAGE gels (7.5% to 10%). Gels were wet transferred using 20% methanol onto nitrocellulose membranes. Membranes were blocked for one hour at room temperature in 5% non-fat milk blocking buffer. Primary antibodies were diluted with 1% non-fat milk in 1× TBST and incubated overnight at 4 °C. Washes were performed with TBS 0.1%Tween-20 (TBST) before the addition of secondary antibody for 1 h at room temperature. Washes were performed with 1× TBST, and the protein detection was performed using Odyssey Infrared imaging system (application software version 3.0.30). Details of primary antibodies are described in Section 2.1.

### 2.6. Luciferase Assays

Cells in 6-well plates were harvested after 48 h of treatment, washed twice in phosphate-buffered saline (PBS), and then lysed in 100–200 μL of 1× Passive Lysis Buffer (Promega, Madison, WI, USA) for 30 min at room temperature (RT). The firefly luciferase activity was analyzed by luciferase reporter assay system (Promega) and normalized by protein concentration of cell lysate.

### 2.7. Transfection

For generating vesicular stomatitis virus G-pseudotyped HIVs, the 293T cells were transfected with plasmids using lipofectamine (Invitrogen/ThermoFisher Scientific, Waltham, MA, USA), applying a previously described methodology [62,63]. The viral titer was determined by the infection of 1 × 10^6^ Jurkat T cells with serial dilutions of the harvested culture supernatant. However, for the transfection of small interfering RNA (siRNA), we used lipofectamine RNAiMAX (Invitrogen/ThermoFisher Scientific), according to the manufacturer’s protocols. For each gene, 4 siRNA target sequences (20 nM each) were used (Table 1). CBF-1 shRNA constructs: besides using Clone ID TRCN0000016204, TRCN0000016203 (Open Biosystems), we also used the following shRNA sequences to clone in a lentiviral vector, and expressed through H1 promoter. As control, cells were either infected with lentiviral vector expressing scrambled shRNA or transfected with a neutral scrambled siRNA sequence. Briefly, the cells were incubated with lipofectamine RNAiMAX reagent and siRNA in opti-MEM medium (Invitrogen/ThermoFisher Scientific) at RT for 5 to 7 min. Subsequently, siRNA-lipid complex was added to the cells and incubated for 48 h at 37 °C in cell culture incubator. Three independent experiments were performed (error bars = SD; *n* = 3). The knockdown effects were assessed by Western blotting using respective antibodies.

### 2.8. Flow Cytometry

The expression of fluorescent reporter gene was assessed through fluorescence-activated cell sorting (FACS) using a FACS Calibur flow cytometer. At 48 h post-stimulation via α-CD3/-CD28 antibody along with IL-2, the expression of fluorescent protein was assessed. The mixed populations were sorted by flow cytometry to enrich 100% HIV-infected cells based on fluorescent protein expression. The shutting down process of latently infected cells to become latent was assessed by flow cytometry every other week [24]. The presence of latent provirus was confirmed by activating the latently infected cells with α-CD3/-CD28 antibody along with IL-2 for roughly 50 h. For some experiments, in order to analyze cells afterword, the cells were fixed with 3% formaldehyde and stored at 4 °C before flow cytometry.

### 2.9. Cell Cytotoxicity: MTS Assay

The cytotoxicity of EZH2, inhibitor DZNep in Jurkat, carrying pHR’-PNL-Luc, was assessed using 3-(4,5-dimethylthiazol-2-yl)-5-(3-carboxymethoxyphenyl)-2-(4-sulfophenyl)-2H-tetrazolium (MTS) reagent (Promega), following the supplier’s protocol. Briefly, the cells were seeded (3 × 10^3^ cells/well) in 96-well plates and incubated with increasing concentrations of DZNep for 2 days. Then, the cells were incubated for 4 h with the MTS reagent directly added in the culture wells. Subsequently, the optical density was measured at 490 nm, using a visible light 96-well plate reader.

### 2.10. Statistical Analysis

Data were analyzed using Microsoft Excel or GraphPad Prism 5.0 (GraphPad Software, San Diego, CA, USA). For paired samples, statistical analyses were performed using Student’s *t* test. One-way analysis of variance (ANOVA) was performed for multiple data point comparisons. Experimental data are presented as the mean ± SD of at least three independent experiments. *p* < 0.05 was considered significant: *p* values were defined as * *p* < 0.05, ** *p* < 0.01 and *** *p* < 0.001.

## 3. Results

### 3.1. CBF-1 Knockdown Disrupts the Latency Maintenance and Leads to the Proviral Reactivation in Primary T Cells

**Figure 1:**
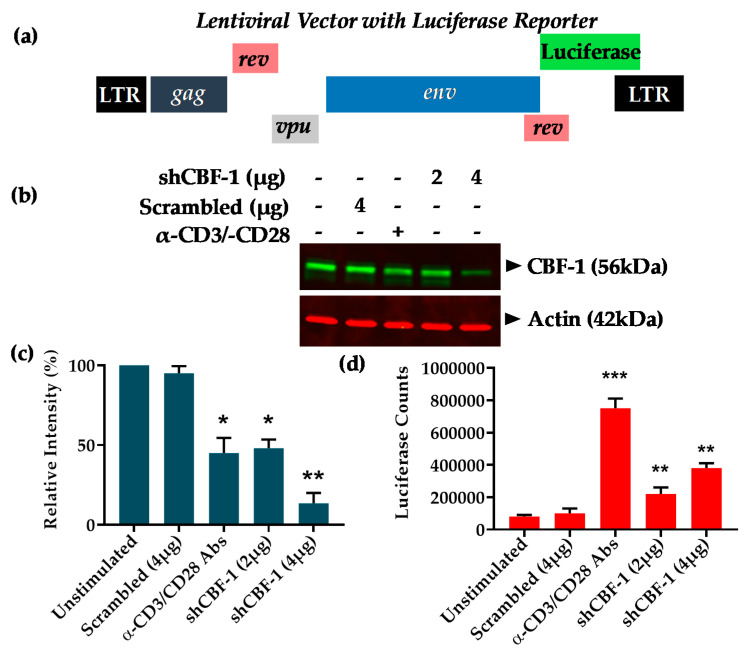
Knockdown of C-promoter binding factor-1 (CBF-1) in primary CD4+ T cells reactivates latent human immunodeficiency virus (HIV) proviruses. (**a**) Structure of lentiviral vector (pHR’-PNL-Luc), which carries reporter luciferase gene under HIV LTR promoter. (**b**) Western blot demonstrating CBF-1 knockdown in cells expressing shRNAs against CBF-1, cells expressing scrambled shRNA and control unstimulated cells. (**c**) Densitometric analyses of immunoblot bands using ImageJ software, and represented graphically after normalization to actin. (**d**) Luciferase assay showing proviral reactivation in primary cells with pHR’-PNL-Luc that are superinfected with different amounts of lentiviral vectors expressing either shRNAs against CBF-1, scrambled shRNA and control unstimulated cells. Error bars represent the Mean ± SD of three independent and separate experiments. The *p* value of statistical significance was set as; *p* < 0.05 (*), 0.01 (**) or 0.001 (***).

We have already confirmed the important role of CBF-1 during the establishing phase of HIV latency, including in primary CD4+ T cells [20,24]. There is an inverse correlation between cellular levels of CBF-1 and HIV gene expression. Accordingly, cells harboring latent provirus have higher levels of CBF-1. However, upon cell activation, we observed a sharp decline in the cellular levels of CBF-1 and a corresponding reactivation of latent provirus. Moreover, using Jurkat cells, a T cell line, the important role of CBF-1 during the maintenance phase of HIV latency was illustrated (Supplementary Figure S2) [20]. In order to extend those studies and further define the role of CBF-1 in primary CD4+ T cells during latency maintenance, we performed some knockdown experiments. We knocked down the endogenous CBF-1 in physiologically relevant primary CD4+ T cells carrying latent provirus, and the reactivation of latent provirus was assessed. The rationale behind doing these experiments was that, if CBF-1-imposed transcriptional restrictions play an essential role in maintaining HIV latency, then its removal should relieve those restrictions and lead to proviral reactivation. The latently infected primary CD4+ T cells harboring pHR’-PNL-Luc HIV provirus, which expresses luciferase as reporter through LTR promoter (Figure 1a), were generated using established methodology [20,24].

To knockdown endogenous CBF-1, the latently infected primary cells were superinfected with lentiviral vectors expressing shRNAs either against CBF-1, or control scrambled shRNA. Scrambled shRNA was confirmed for its neutrality towards HIV and cellular genomes using the NCBI program Blast [20]. More than 70% knockdown (** *p* < 0.01) was obtained using 4 μg of shRNA vectors (Figure 1b,c). Depletion of CBF-1 resulted in significant reactivation of latent provirus (** *p* < 0.01), indicated by the enhanced expression (more than three-fold) of luciferase reporter gene compared to a scrambled shRNA control and unstimulated cells (Figure 1d). As positive control to show the population of cells carrying reactivable latent provirus in their genome, cells were activated through T cell receptor (TCR) stimulation by treating the cells with anti-CD3/-CD28 antibodies in the presence of IL-2 (α-CD3/CD28/IL-2).

Following TCR stimulation, we noted more than double the luciferase counts than those obtained upon CBF-1 knockdown. The results were also reproduced in cells that express GFP as reporter. As detailed above, we also observed the reduction of cellular levels of CBF-1 following cell activation via TCR stimulation (Figure 1b,c). Partial reactivation of latent provirus after CBF-1 depletion indicated the involvement of additional factors in restricting HIV gene expression during the maintenance phase of HIV latency. Moreover, besides epigenetic restrictions, other mechanisms also play role in restricting HIV in the latent state [9,12,15,65]. These results in primary T cells along with our previously published data using T cell lines [20] verified the important role of CBF-1 during the maintenance phase of HIV latency. Hence, CBF-1 besides inducing the establishment of HIV latency [20], promotes the maintenance of HIV latency.

### 3.2. CBF-1 Recruited PRCs Play Direct Role in Sustaining HIV Provirus in Latent State

**Figure 2:**
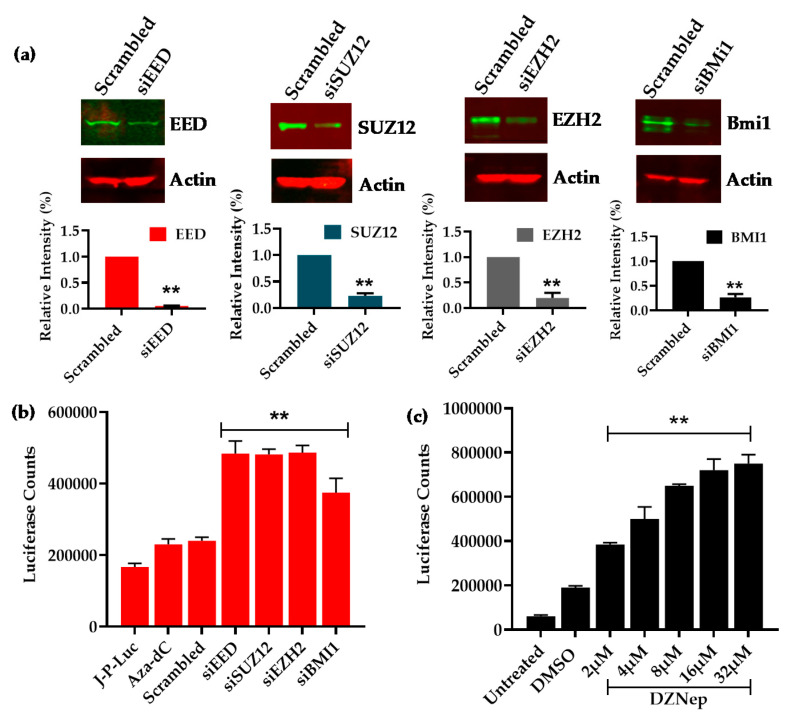
Knockdown of polycomb group (PcG) complex led to proviral reactivation. Some of the core PcG complex components were knocked down individually by transfecting latently infected Jurkat-pHR’-PNL-Luc cells with four specific siRNAs. HIV-1 reactivation of latent provirus was quantified through luciferase assays performed after 52 h either post siRNA transfection or 48 h post DZNep treatment. (**a**) Western blot showing the efficiency of siRNA to knockdown indicated subunits of PRCs. The densitometry analyses were then represented graphically after normalization to actin. Quantitative luciferase assays marking proviral reactivation either after (**b**) knockdown of individual subunits belonging to PRCs or (**c**) upon DZNep treatment (from 2 µM to 32 µM) of cells. Graphs represent the average and standard deviation from three independent and replicate samples. Statistical analysis was done using Microsoft Excel and GraphPad Prism 5.0 (GraphPad Software, San Diego, CA, USA). The *p* value of statistical significance was set as: *p* < 0.01 (**).

In order to establish the direct role of the PRCs in controlling HIV latency, we knocked down the core components of both PRC1 and PRC2. Subsequently, we examined whether the removal of repression posed by PRCs on HIV transcription leads to the reactivation of the latent HIV proviruses. Jurkat cells harboring latent HIV provirus pHR’-PNL-Luc, a lentiviral vector carrying the luciferase reporter gene under the control of the HIV LTR (Figure 1a), were used. The cells were transfected with the 20 nM siRNA against main components of PRCs (PRC1 and PRC2). We used a mixture of four siRNAs (20 nM each) against each target gene, and the non-targeting scrambled siRNA was used as control. When compared with scrambled control, a significant knockdown of more than 70% (** *p* < 0.01) was evident for each target gene (Figure 2a). The reactivation of latent provirus was assessed through luciferase reporter assay. Around three-fold reactivation of latent provirus was observed following knockdown of each component of PRCs (** *p* < 0.01) (Figure 2b). Notably, the knockdown of the components of PRC2 was slightly more effective than PRC1 subunits in reactivating latent proviruses. A similar effect with the knockdown of each target gene suggested the removal of any of the core component of PRCs destabilizes the corepressors complex at LTR.

Similar results were obtained when we disabled the PRC2 complex by inhibiting the EZH2 using DZNep, a broad-spectrum histone methylation inhibitor. DZNep is known to downregulate the cellular levels of several histone methylases, mainly EZH2 [66]. Latently infected Jurkat cells harboring the latent HIV provirus, pHR’-PNL-Luc, were treated dose-dependently with DZNep (2 μM to 32 μM). After 48 h, cell extracts were assessed for the activity of luciferase enzyme by performing luciferase assays. As anticipated, the inhibition of PRC2 by DZNep led to proviral reactivation (** *p* < 0.01), which further validates the vital role of PRC2 in promoting HIV latency (Figure 2c). Additionally, these results were further reproduced in another latently infected T cell line (2D10 cells), which expresses GFP as reporter (Supplementary Figure S3).

To exclude the possibility that the loss of luciferase activity was due to non-specific cell cytotoxicity of DZNep (EZH2 inhibitor), cells were treated with increasing dose of inhibitor and cell viability was assessed using a MTS assay. We did not find any significant cell cytotoxicity up to the dose of 32 μM even after 5 d in culture (Supplementary Figure S3a). More than three-fold proviral reactivation was observed at concentrations of 8 μM and beyond. At doses higher than 8 μM, little proviral reactivation was observed (Supplementary Figure S3b,c). These results further corroborated the direct role of PRCs in restricting the transcription of latent HIV provirus.

### 3.3. CBF-1 Promotes HIV Latency by Inducing Multiple Types of Repressive Epigenetic Modifications at HIV LTR

In earlier publications, we demonstrated that CBF-1 restricts HIV transcription by recruiting HDACs containing corepressor complexes at HIV LTR. HDACs subsequently mediate the deacetylation of core histones, eventually facilitating the establishment of HIV latency, both in transformed and primary CD4+ T cells [20,24]. Since various corepressor complexes contain HDACs, a goal was to define the precise identity of the corepressor complex recruited by CBF-1 at HIV LTR during HIV latency. In addition to histone deacetylation, numerous studies including ours have established the importance of other repressive epigenetic modifications, such as the tri-methylation of core histone H3 at position 9 (H3K9me3) and 27 (H3K27me3) during HIV latency [9,20,24,28,29,67]. The role of enzymes responsible for catalyzing these epigenetic modifications during HIV latency has also been well documented [28,29,30,31,52,68]. We have also confirmed the role of H3K9me3 and H3K27me3 in primary CD4+ T cells during HIV latency [24]. We investigated whether CBF-1-recruited corepressors are responsible for inducing those repressive histone H3 methylations and promoting HIV latency. In order to determine whether CBF-1 is responsible for inducing varying repressive epigenetic modifications, we assessed the impact of CBF-1 knockdown on the resultant epigenetic changes at HIV LTR. If the enzymes present in the CBF-1 recruited corepressor complex are responsible for catalyzing H3K9me3 and H3K27me3 modifications, then CBF-1 knockdown should result in the reduced recruitment of the corepressor complex and thus, less generation of H3K9me3 and H3K27me3 at HIV LTR.

To investigate this latently infected Jurkat T cell line, a clone E4, in which the Nef gene is replaced by a short lived green fluorescent protein (d2EGFP) reporter, was used [20,31]. The latently infected cells were superinfected with lentiviral vectors carrying shRNAs, either against CBF-1 or control scrambled shRNA, a target neutral sequence. The cellular population carrying shRNA vectors was enriched via puromycin selection.

**Figure 3:**
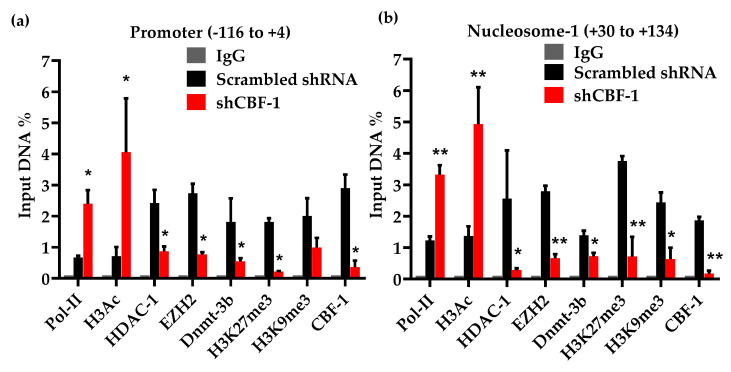
CBF-1 restricts HIV transcription by inducing multiple types of repressive epigenetic modifications at HIV LTR. Chromatin immunoprecipitation (ChIP) analyses were performed using latently infected Jurkat T cells to evaluate the turnover of different epigenetic modifications at HIV LTR in the absence or presence of knockdown of endogenous CBF-1, using the indicated antibodies. Primer sets directed to the (**a**) Promoter region (−116 to +4) with respect to transcription start site; (**b**) Nucleosome 1 (+30 to +134) with respect to transcription start site of HIV-1 LTR. The depicted ChIP assay results were reproduced 5 times. Graphs represent the average and standard deviation from three independent and replicate samples. Statistical analysis was calculated with GraphPad Prism 5.0 (GraphPad Software, San Diego, CA, USA). The *p* value of statistical significance was set at either; *p* < 0.05 (*) or 0.01 (**).

To assess the binding of different transcription and epigenetic factors, besides changes in corresponding epigenetic modifications at HIV LTR in the absence or presence of CBF-1 knockdown, quantitative ChIP assays were performed, and the two critical regions of HIV LTR, promoter (Figure 3a) and nucleosome-1 (Nuc-1) (Figure 3b) were examined. The immunoprecipitated DNA was measured through q-PCR using primer sets directed to the promoter region (−116 to +4) and nucleosome-1 region (+30 to +134) of LTR with respect to the transcription start site. To provide a control for equal loading, the results were normalized with housekeeping GAPDH gene expression (−145 to +21), a constitutively expressed cellular gene. Latently infected Jurkat cells showed low levels of RNAP II at both the promoter and Nuc-1 regions of LTR (Figure 3). Given ChIP resolution capacity of ~500 bp due to DNA shearing limit during sonication, we found overlapping signals at the promoter and neighboring Nuc-1 regions of LTR. Nevertheless, histone modifications were clearly more prevalent at the histone-rich Nuc-1 region (Figure 3b).

The lower amount of RNAP II at LTR of latent provirus confirms that latent HIV proviruses are restricted in HIV transcription. As anticipated, we found higher levels of CBF-1 and HDAC-1 at latent provirus, in accordance with our previous studies that demonstrated that after binding to LTR, CBF-1 recruits HDACs containing corepressor complexes [20]. We also observed the accumulation of other heterochromatic marks, H3K9me3 and H3K27me3, at HIV LTR of latent provirus. This observation verified that CBF-1 promotes the transcriptional silencing of latent provirus by inducing multiple layers of repressive epigenetic modifications at HIV LTR. Interestingly, following CBF-1 knockdown, the level of CBF-1 at HIV LTR drops sharply, confirming that there is a lower amount of CBF-1 in the cell for recruitment to LTR. As anticipated, we found parallel dissociation of HDACs containing corepressor complexes from LTR, demonstrated by the removal of HDAC-1, further illustrating the direct role of CBF-1 in recruiting HDACs. The loss of HDACs resulted in the enhanced acetylation of core histones, represented by the hyperacetylation of core histone H3 following CBF-1 knockdown. Notably, there is a corresponding loss of other repressive epigenetic modifications from LTR following CBF-1 knockdown, namely H3K9me3 and H3K27me3. This indicates that the enzymes present in the CBF-1 recruited corepressor complex are responsible for catalyzing these epigenetic modifications. In fact, following CBF-1 knockdown, we found a corresponding loss of EZH2 (** *p* < 0.01) from LTR, an enzyme that catalyzes H3K27me3, and the core component of PRC2 corepressor complex. On the other hand, the establishment of the epigenetic mark H3K9me3 is usually catalyzed by SUV39H1 and G9A [32]. The presence of SUV39H1 and G9A at HIV LTR and their role during HIV latency have been well documented [29,30,54,69]. Both SUV39H1 and G9A are known to interact with various subunits of PRC1 [40,70], suggesting the presence of PRC1 as well. The histone methylation has been shown to promote the recruitment of DNA methyltransferases [71]; accordingly, we found the corresponding loss of Dnmt-3b following CBF-1 knockdown. The presence of different DNA methyltransferases in PRC complexes is well documented [72,73]. Together, these findings suggested that, along with PRC2, CBF-1 brings PRC1 to HIV LTR during HIV latency.

### 3.4. CBF-1 Promotes Both the Establishment and the Maintenance of HIV Latency by Recruiting PRCs at HIV LTR

**Figure 4:**
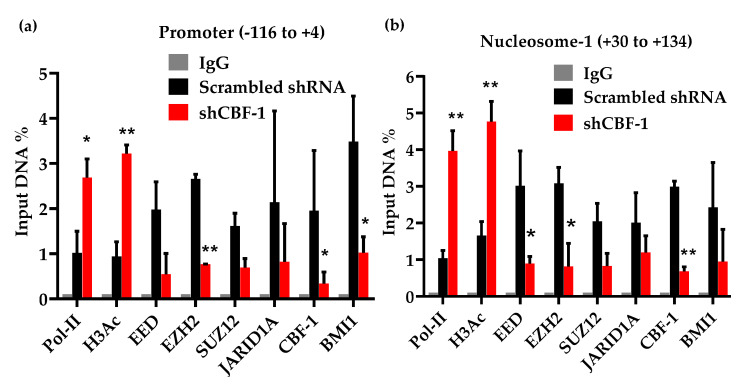
CBF-1 knockdown resulted in dissociation of different factors belonging to both PRCs (PRC1 and PRC2). ChIP analyses were performed using latently infected Jurkat T cells in the absence or presence of CBF-1 knockdown. CBF-1 knockdown leads to the dissociation of various core components of both PRCs, showing the role of CBF-1 in their recruitment at HIV LTR. (**a**) Promoter region (−116 to +4); (**b**) Nucleosome 1 (+30 to +134). Error bars represent the SEM of three independent experiments and three separate qPCR measurements from each experiment. Graphs represent the average and standard deviation from three independent and replicate samples. Statistical analysis was calculated with GraphPad Prism 5.0 (GraphPad Software, San Diego, CA, USA). The *p* value of statistical significance was set at either; *p* < 0.05 (*) or 0.01 (**).

The presence of PRC2 at HIV LTR and its role during HIV latency establishment and maintenance have been well documented [52,53,54,55]. However, factors involved in the recruitment of PRCs at LTR are not fully defined. We investigated if CBF-1 is one of the factors that promote the recruitment of PRCs at HIV LTR. We evaluated the recruitment profile of the main core components of PRC1 and PRC2 corepressor complexes at HIV LTR, before and after knocking down the endogenous CBF-1 protein. The levels of different factors were assessed by performing quantitative ChIP assays (Figure 4). The latently infected Jurkat cells were transduced with lentiviruses expressing shRNAs, either against CBF-1 or neutral scrambled shRNA. We determined the recruitment kinetics of different core components belonging to PRC1 and PRC2 corepressor complexes at two critical regions of HIV LTR, promoter and Nuc-1. We detected higher levels of core components of PRC1 and PRC2 complexes at the LTR of latent HIV provirus, namely EED, SUZ12, EZH2 and BMI1 (Figure 3). The detection of EED, SUZ12, and EZH2 marks the presence of PRC2, while the recruitment of BMI1 indicates the presence of PRC1. This result shows that latent provirus accumulates both PRCs at its LTR, and suggests the role of PRCs during HIV latency. However, upon CBF-1 knockdown, we observed the corresponding dissociation of core components of both PRCs complexes from LTR (Figure 4a,b).

The parallel loss of core components of PRC1 and PRC2 following CBF-1 knockdown confirms that CBF-1 is responsible for their recruitment at HIV LTR. Thus, our results convincingly demonstrate that CBF-1 promotes HIV latency by recruiting PRC1 and PRC2 at HIV LTR. The direct interaction of CBF-1 with PRC1 and PRC2 was further validated through a GST-pull down assay (Supplementary Figure S4). We also found the presence of JARID1A at HIV LTR. JARID1A is a histone H3K4me3 demethylase, which removes the methyl group from histone H3 at position 4 (H3K4me3), a euchromatic mark. At cellular promoters, JARID1A has been shown to interact with both CBF-1 and its recruited corepressor complexes, including PRCs [25,74,75]. The accumulation of epigenetic modifications, such as H3Ac and H3K4me3 supports the establishment of transcriptionally active or euchromatin structures. We found that JARID1A and HDACs of PRCs remove these pro-euchromatin modifications (H3K4me3 and H3Ac) at HIV LTR. Therefore, besides harboring the enzymes that induce the formation of transcriptionally repressive heterochromatin structures, PRCs also carry the enzymes which remove the euchromatin structures, consequently supporting prolonged or permanent gene silencing. Hence, by recruiting PRCs at HIV LTR, CBF-1 not only facilitates the establishment of HIV latency, but also promotes the maintenance or stabilization of HIV latency. This conclusion is also supported by the observation that when we knockdown the endogenous CBF-1, the latent provirus gets reactivated (Figure 1). Thus, by recruiting PRCs at HIV LTR, CBF-1 induces multiple layers of repressive epigenetic modifications to form transcriptionally repressive heterochromatin structures at HIV LTR during HIV latency. Consequently, CBF-1 not only promotes, but also stabilizes the silencing of latent proviruses.

### 3.5. CBF-1 Recruited PRCs Facilitate HIV Latency in Primary CD4+ T Cells

Like an ideal repressor of HIV transcription, CBF-1 is present in abundant amounts in resting T cells. However, upon cell activation, CBF-1 levels drop sharply, a property also visible in Figure 1b,c. This unique characteristic of CBF-1 has been confirmed in different cell types [20]. This implies that after cell activation, reduced cellular levels of CBF-1 result in poor recruitment of CBF-1 at LTR. In parallel, if CBF-1 contributes to the recruitment of PRCs at LTR, then we envisioned proportionally reduced recruitment of PRCs at LTR.

**Figure 5:**
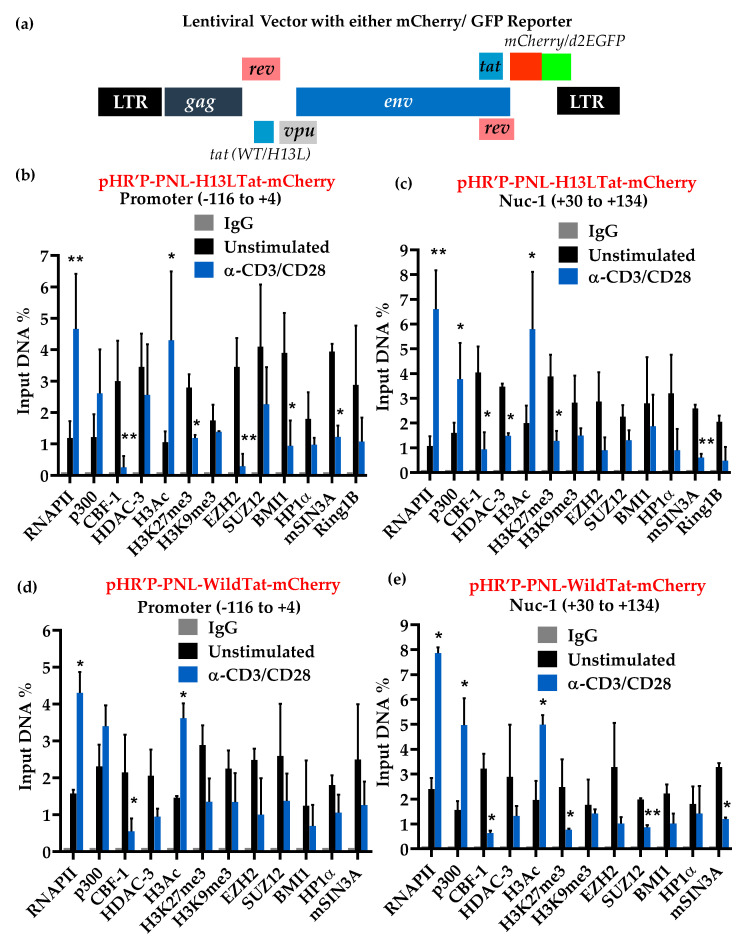
Cell activation leads to fluctuation in the levels of different chromatin-associated factors that belong to PRC1 and PRC2. ChIP analyses were performed before and after activation of latently infected primary CD4+ T cells with α-CD3/-CD28 antibodies, in the presence of IL-2 for 30 min. (**a**) Structure of lentiviral vectors. mCherry was used as reporter depicted in this diagram. ChIP results in latency systems harboring proviruses with the vector pHR’-PNL-H13LTat-mCherry (**b**,**c**), and pHR’-PNL-wild-typeTat-mCherry (**d**,**e**). Error bars represent the SEM of two independent experiments and three separate qPCR measurements from each analysis. Graphs represent the average and standard deviation from three independent and replicate samples. Statistical analysis was calculated with GraphPad Prism 5.0 (GraphPad Software, San Diego, CA, USA). The *p* value of statistical significance was set at either; *p* < 0.05 (*) or 0.01 (**).

Thus, to validate our hypothesis and provide physiological relevance to these findings, we performed ChIP assays using latently infected primary CD4+ T cells (Figure 5). The latently infected primary CD4+ T cells, harboring either pHR’-PNL-H13LTat-mCherry (Figure 5a–c), pHR’-PNL-wildTat-mCherry (Figure 5d,e) or pHR’-PNL-H13LTat-d2GFP (Figure 5), were generated using Tyagi-Sahu primary T cell based latency model, described earlier [24,64] (Supplementary Figure S1a). These HIV-derived vectors (Figure 5a) express fluorescent protein reporter genes (either the short-lived d2EGFP or mCherry) in place of the nef gene, as detailed earlier [20,24,31]. These viruses express the regulatory proteins Tat and Rev. Like complete HIV, the positive feedback circuit that enhances HIV transcriptional elongation and export of mRNA from the nucleus is fully intact. In some of our experiments, in order to increase the frequency of latently infected cells in the population, we utilized Tat carrying the H13L mutation. This partially attenuated Tat variant was originally identified in the U1 latently infected cell line and is highly prevalent in latent proviral pools of HIV patients [76,77]. Quantitative ChIP assays were performed before and after activating the latently infected primary CD4+ T cells with α-CD3/-CD28 antibodies in the presence of IL-2 for 30 min. The immunoprecipitated DNA was measured through q-PCR, using primer sets directed to the promoter region (−116 to +4) and nucleosome-1 region (+30 to +134) of LTR, with respect to the transcription start site. Binding of different transcription factors and epigenetic changes at these regions of LTR dictate the overall rate of HIV transcription. As a control for equal loading in each well, the results were normalized with GAPDH gene expression (−145 to +21), a constitutively expressed cellular gene.

As depicted in Figure 5, lower levels of RNAP II were present at the promoter and Nuc-1 regions of latent provirus, validating highly restricted gene expression from LTR promoter of latent provirus. However, in the case of cells infected with provirus carrying wild-type Tat, we observed comparatively higher levels of RNAP II (Figure 5d,e). The reason behind this anomaly is that this cell population consists of around 70% latently infected cells (Figure 5d,e), compared to around 95% latently population in the case of cells infected with provirus carrying H13L Tat (Figure 5b,c). The overall LTR binding profiles of different factors were quite comparable in case of cells harboring latent provirus either with wild-type or H13L Tat. We found higher levels of CBF-1, its binding partner mSIN3A and HDAC-1 and -3 at latent proviral LTR in primary T cells. In parallel, we found that the LTR of latent HIV contains stable heterochromatin structures, indicated by the higher presence of histone H3 deacetylation, H3K9me3 and H3K27me3. Analogous to transformed T cells, we found higher recruitment of both PRC1 (BMI1 and RING1) and PRC2 (EZH2 and SUZ12) at the latent HIV LTR (Figure 5).

These findings validate the vital role of PRCs during HIV latency in primary T cells. Reduced levels of RNAP II marks restricted ongoing HIV transcription from LTR. However, TCR stimulation, a condition that results in reactivation of latent provirus [24], led to a five- to seven-fold increase in RNAP II levels at both promoter and Nuc-1 regions. Higher recruitment of RNAP II marks enhanced ongoing HIV transcription after TCR stimulation. Concomitantly, we also observed a substantial loss of CBF-1 and recruited corepressor complex, PRCs, from LTR, indicated by the loss of mSIN3A, HDAC-1, HDAC-3, EZH2, SUZ12, BMI1 and RING1B (Figure 5). Loss of HDACs from LTR translated into enhanced acetylation of core histones, indicated by a four to seven-fold increase in the acetylation of histone H3 present at the LTR. Similar to our previous observations in primary T cells [24], we found the removal of repressive H3K9me3 and H3K27me3 heterochromatic marks from LTR, recruitment of histone acetyltransferase, p300 at HIV LTR following TCR stimulation. As noted earlier in primary T cells [24], we found enhanced recruitment of NF-κB (p65) at the promoter region of LTR, as NF-κB binding sites reside in that region (data not shown).

Given the ChIP resolution limit of ~500 bp, an overlap of signals between adjacent regions, such as promoter and Nuc-1, was expected. Therefore, to some extent, we observed similar histone changes at both LTR regions. Nevertheless, a notable difference in the levels of histone modifications was clearly visible in the Nuc-1 region of the proviruses. These results are consistent with previous studies using transformed cell lines, which have shown that the HIV promoter region is relatively devoid of histones [9,20,24,78,79]. In summary, these results have shown that CBF-1 restricts HIV transcription by recruiting both PRC1 and PRC2 during HIV latency in primary CD4+ T cells. We thus validated the role of CBF-1 and PRCs during both the establishment and the maintenance of HIV latency in primary CD4+ T lymphocytes.

## 4. Discussion

In our previous studies, we demonstrated that CBF-1, after binding to its cognitive sites at HIV LTR, strongly and selectively represses HIV transcription. In this paper, we show that CBF-1 promotes the establishment and maintenance of HIV latency by recruiting Polycomb corepressor complexes at HIV LTR. The polycomb group (PcG) proteins are divided in the form of two main corepressor complexes, PRC1 and PRC2 [80], that we showed to be present at the HIV LTR. PRCs, by inducing transcriptionally repressive epigenetic modifications, facilitate the assembly of heterochromatin structures at HIV LTR. The PRC1 complex mainly catalyzes the monoubiquitination of histone H2A at lysine 119 residue (H2AK119Ub1) through its Ring subunits, Ring1A/B, which contain E3 ligase activity a [41,81,82]. On the other hand, PRC2 is primarily characterized by the presence of the histone methyltransferases EZH1/2, which, along with other subunits, mainly SUZ12 and EED, catalyze the di- or trimethylation of histone H3 at lysine 27 (H3K27me2/3) [34,82]. Given the fact that CBF-1 expression is strongly reduced following T-cell activation, it is expected that TCR activation in CD4 T-cells should reflect the results obtained after CBF-1 knockdown. In our previous investigations, we showed the important role of CBF-1 during HIV latency, by performing experiments in transformed T cell lines [9,20]. Here, we have extended those findings and confirmed the significant role of CBF-1 during HIV latency in physiologically relevant primary CD4+ T cells.

The role of H3K9me3 and H3K27me3 during HIV latency is well established. Moreover, we have shown the importance of these repressive epigenetic marks during HIV latency in primary CD4+ T cells [24]. The formation of the epigenetic mark H3K27me3 is mainly catalyzed by EZH2 enzyme. We noted higher levels of EZH2 and deposition of H3K27me3 at the LTR of latent provirus present in primary T cells. EZH2 is the core component of PRC2, and Karn’s group has convincingly demonstrated the presence and role of EZH2 and of PRC2 during HIV latency establishment and maintenance [52,54]. These results have been validated by other groups [53,55,83]. However, the identity of the factor(s) that recruit PRCs at HIV LTR and promote HIV latency were not well defined. Recently, Karn’s group has demonstrated the role of JARID2 in recruiting PRC2 at HIV LTR [54]. In a similar manner, we have been investigating the role of CBF-1 as a recruiter of PRCs at LTR. We proposed that, if CBF-1 is responsible for the recruitment of PRC2 and EZH2, then CBF-1 reduction at LTR should translate to the lesser accumulation of PRC2 and of H3K27me3 at HIV LTR. Accordingly, we found the comparable loss of EZH2 and H3K27me3 from LTR upon CBF-1 knockdown (Figure 3). In later CBF-1 ablation experiments, we observed the corresponding loss of other core components of PRC2, namely EED and SUZ12, from LTR (Figure 3 and Figure 4). Altogether, these results confirmed the direct role of CBF-1 in recruiting PRC2 at HIV LTR during HIV latency.

Upon CBF-1 knockdown, we found a parallel loss of the epigenetic mark H3K9me3, suggesting that the CBF-1 recruited corepressor complex also carries the enzyme that catalyzes the H3K9me3 epigenetic modification. Notably, PRC2 does not carry any enzyme that catalyzes H3K9me3, but PRC1 is known to bring SUV39H1 and G9A along with it [40,70]. SUV39H1 and G9A are the two main enzymes which catalyze the formation of the epigenetic mark H3K9me3 at nucleosomes. This observation suggested that, along with PRC2, CBF-1 also brings PRC1 to HIV LTR for the generation of transcriptionally repressive heterochromatin structures at HIV LTR during viral latency. In fact, we observed a comparable loss of BMI1 and RING1B, two core components of PRC1, following CBF-1 knockdown in both T cell line and primary T cells (Figure 4 and Figure 5). The presence of PRC1 at HIV LTR during latency has also been noted by other investigators [52,54,55]. However, the factor that brings PRC1 at LTR was not known until our investigation demonstrated that CBF-1 is the cellular protein, which, after binding to LTR at specific sites, brings both PRC1 and PRC2 to inhibit HIV transcription during HIV latency.

Following CBF-1 knockdown, we observed the parallel loss of Dnmt-3b, a DNA methyltransferase. Dnmt-3b has been shown as a component of various PRC complexes. This finding further validated that CBF-1 promotes HIV latency by inducing multiple layers of repressive epigenetic modifications, via recruiting different PRC complexes at HIV LTR [71,72,73]. In our earlier investigations, we showed that, in resting T cells that harbor latent provirus, higher levels of CBF-1 are present. However, upon cell activation, the cellular level of CBF-1 drops sharply and latent HIV proviruses get reactivated [20]. Thus, CBF-1 acts as an ideal transcriptional repressor which plays a vital role in regulating HIV latency. Therefore, to further validate that cell activation leads to the decline of cellular CBF-1 levels, we showed the lesser recruitment of CBF-1 at LTR, and consequently, the loss of PRCs from LTR upon cell activation.

The physiological relevance of these findings is evident, since they were reproduced in latently infected primary CD4+ T cells. We validated the presence of PRCs at latent HIV proviruses and confirmed their removal from HIV LTR upon cell stimulation through TCR induction (Figure 5). Proviral reactivation was indicated by the higher RNAP II recruitment and confirmed through the enhanced expression of the reporter gene luciferase. Using latently infected primary CD4+ T cells in an earlier study, we demonstrated the presence of both H3K9me3 and H3K27me3 at the LTR of latent provirus, which drops sharply following TCR stimulation [24,82]. Similar to our previous findings, we noted the presence of components of both PRCs, representing the presence of PRC1 and PRC2 at latent provirus, which is abruptly dissociated from HIV LTR upon TCR stimulation (Figure 5).

Notably, besides core components of PRCs, we also found the presence of certain interacting partners or auxiliary factors of PRCs, such as HDACs, mSIN3A and HP1α [38]. These factors either bind directly to PRCs components or to the induced epigenetic modifications (e.g., H3K9me3 modification promotes the recruitment of HP1 proteins). Subsequently, to confirm the direct role of PRCs during HIV latency, we assessed the reactivation of latent provirus after the knockdown of different core components of PRC1 and PRC2 repressor complexes (Figure 2). If PRCs play a significant role in the silencing of latent HIV provirus, then their removal or reduction by knockdown should relieve that restriction and lead to proviral reactivation. Consistent with this idea, upon knockdown of the core components, PRCs become destabilized, and we observed the two- to three-fold reactivation of latent provirus (Figure 2). Notably, we observed better proviral reactivation following the knockdown of PRC2 components than PRC1, suggesting a primary role of PRC2 components in the stability of corepressor complex recruited by CBF-1. Supporting this observation, it has been documented that PRC2/EZH2-induced H3K27 methylation promotes the recruitment of PRC1 to target cellular genes, and the disruption of PRC2 leads to the loss of PRC1 from chromatin targets, but the other way around is not always that effective [84]. Interestingly, upon CBF-1 knockdown, we found the corresponding loss of JARID1A, an enzyme which is known to interact with PRC subunits [25,74]. JARID1A is a histone demethylase that selectively demethylates the histone H3 at position K4, H3K4me3. In contrast to the above-mentioned trans-repressive epigenetic changes in H3K9me3 and H3K27me3, the trimethylation of histone H3 at lysine 4 (H3K4me3) is a euchromatic mark, an epigenetic modification that promotes the establishment of a transcriptionally active euchromatin structure which supports transcription. Thus, JARID1A, by removing H3K4me3, inhibits the generation of the euchromatin structure at LTR. Consequently, certain enzymes that are recruited by PRCs, such as JARID1A and HDACs, remove euchromatic marks, namely H3K4me3 and acetylation of histones, respectively, to provide stability to gene silencing and restrict transient gene reactivation. Hence, in addition to promoting the establishment of latency by recruiting PRCs at HIV LTR, CBF-1 promotes the maintenance or stabilization of HIV latency. Moreover, the reactivation of latent provirus following CBF-1 knockdown or TCR stimulation further validates the role of CBF-1 during the maintenance phase of HIV latency. Both CBF-1 knockdown or cell activation reduce cellular CBF-1 levels. Therefore, when we removed the restriction posed by CBF-1 through knocking it down or via TCR stimulation, the latent provirus becomes reactivated (Figure 1 and Figure 5).

**Figure 6:**
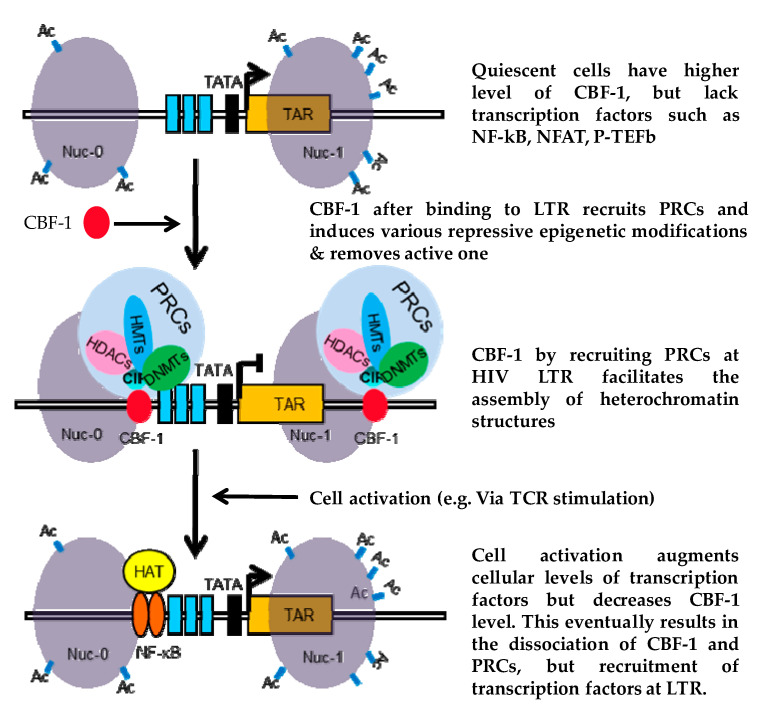
Model of CBF-1 functioning. Based on our findings, we propose the following model for the regulation of HIV latency by CBF-1. The higher levels of CBF-1 and lack of transcription factors such as NF-kB and NFAT in quiescent cells facilitates the binding of CBF-1 at HIV LTR. CBF-1 after binding to LTR recruits PRCs. PRCs subsequently promote heterochromatin environment at HIV LTR and inhibit the free flow of transcription machinery, thus facilitating the establishment and maintenance of HIV latency. Following cellular activation, the levels of CBF-1 drop, but the levels of NF-kB and NFAT rise in the nucleus, which displaces CBF-1 and corepressor complexes from their binding sites. Eventually, these factors recruit coactivator complexes at HIV LTR, which then establishes the euchromatin environment at HIV LTR that facilitate the access of transcription machinery at the LTR promoter, and thus leads to the reactivation of latent proviruses.

To summarize our results, we propose a model to depict the role of CBF-1 in restricting HIV transcription during latency (Figure 6). According to our model, in the absence of transcription factors such as NF-kB and NFAT in quiescent cells, CBF-1 binds to the specific sites at HIV LTR and recruits PRCs. Enzymes of the PRCs subsequently induce multiple layers of repressive epigenetic modifications and remove transcriptionally active epigenetic modifications. These epigenetic changes subsequently facilitate the generation of transcriptionally repressive heterochromatin structures at HIV LTR. Heterochromatin structures restrict the free flow of transcription factors at HIV LTR, which eventually restrict HIV transcription and stabilize restriction. Thus, CBF-1 facilitates the establishment and maintenance of HIV latency. Following cell activation, the levels of CBF-1 drop, whereas levels of NF-kB, NFAT, and other transcription factors rise in the nucleus, displacing CBF-1 and PRCs from LTR. Successively, these factors recruit coactivator complexes at HIV LTR, which then establish the euchromatin environment at HIV LTR, facilitating the access of transcription machinery at LTR promoter, and thus leading to the reactivation of latent proviruses. Taken together, our results validated that CBF-1 suppresses HIV gene expression, by recruiting both PRC1 and PRC2 at HIV LTR. Hence, we conclude that, by recruiting PRCs, CBF-1 facilitates both the establishment and maintenance phases of HIV latency.

## 5. Conclusions

In conclusion, our study confirms that CBF-1 promotes both the establishment and maintenance of HIV latency in primary T cells, by recruiting PRC1 and PRC2 at HIV LTR. From a clinical standpoint, our findings suggest that for the reactivation of latent proviruses, targeting factors that recruit those enzymes at HIV LTR, which will result in more profound reactivation of latent provirus than targeting individual enzymes that induce repressive epigenetic modifications. In fact, the removal of the whole corepressor complex will relieve multiple repressive epigenetic modifications simultaneously, and could prove to be a better latency reversing strategy, a prerequisite for viral eradication. Through in vitro studies, we found that PRC2 complex prefers naked DNA for binding over histone-studded chromatinized DNA structures [85]. However, DNA rarely presents as naked in vivo, as chromatinization takes place soon after its synthesis. Thus, our findings suggest that besides direct recruitment of PRC through CBF-1-PRC subunit(s) interactions, CBF-1 binding may create some necked space in DNA that allows better recruitment of PRC. This will be the focus of our future investigations.

## Figures and Tables

**Table 1:** List of sequences of siRNA, shRNA and constructs.

Name of Target Gene	Sequence (5′-3′)
SUZ12	GCTGACAATCAAATGAATCAT
	CCAAACCTCTTGCCACTAGAA
	GCTTACGTTTACTGGTTTCTT
	CGAAACTTCATGCTTCATCTA
EED	GACACTCTGGTGGCAATATTT
	CCTATAACAATGCAGTGTATA
	GTGCGATGGTTAGGCGATTTG
	CTGGATCTAGAGGCATAATTA
EZH2	CGGCTCCTCTAACCATGTTTA
	CCCAACATAGATGGACCAAAT
	GCTGACCATTGGGACAGTAAA
	CAACACAAGTCATCCCATTAA
BMI1	ATTGATGCCACAACCATAATA
	GGAACCTTTAAAGGATTATTA
	CAGCAAGTATTGTCCTATTTG
	TAATGGATATTGCCTACATTT
Scrambled	TTGATGCACTTACTAGATTAC

## Supplementary Materials

Supplementary materials can be found at https://www.mdpi.com/1999-4915/12/9/1040/s1.
