# Supplementary material for: CBF-1 Promotes the Establishment and Maintenance of HIV Latency by Recruiting Polycomb Repressive Complexes, PRC1 and PRC2, at HIV LTR
Source: Viruses. 2020 Sep 18;12(9):1040. doi: 10.3390/v12091040 (PMC7551090; doi:10.3390/v12091040)
Supplement: Supplementary file 1 [file viruses-12-01040-s001.zip › Sharma et al Supplementary files.pdf]

CBF-1 promotes the establishment and maintenance of HIV latency by recruiting Polycomb repressive complexes, PRC1 and PRC2, at HIV LTR

Supplementary Figure 1

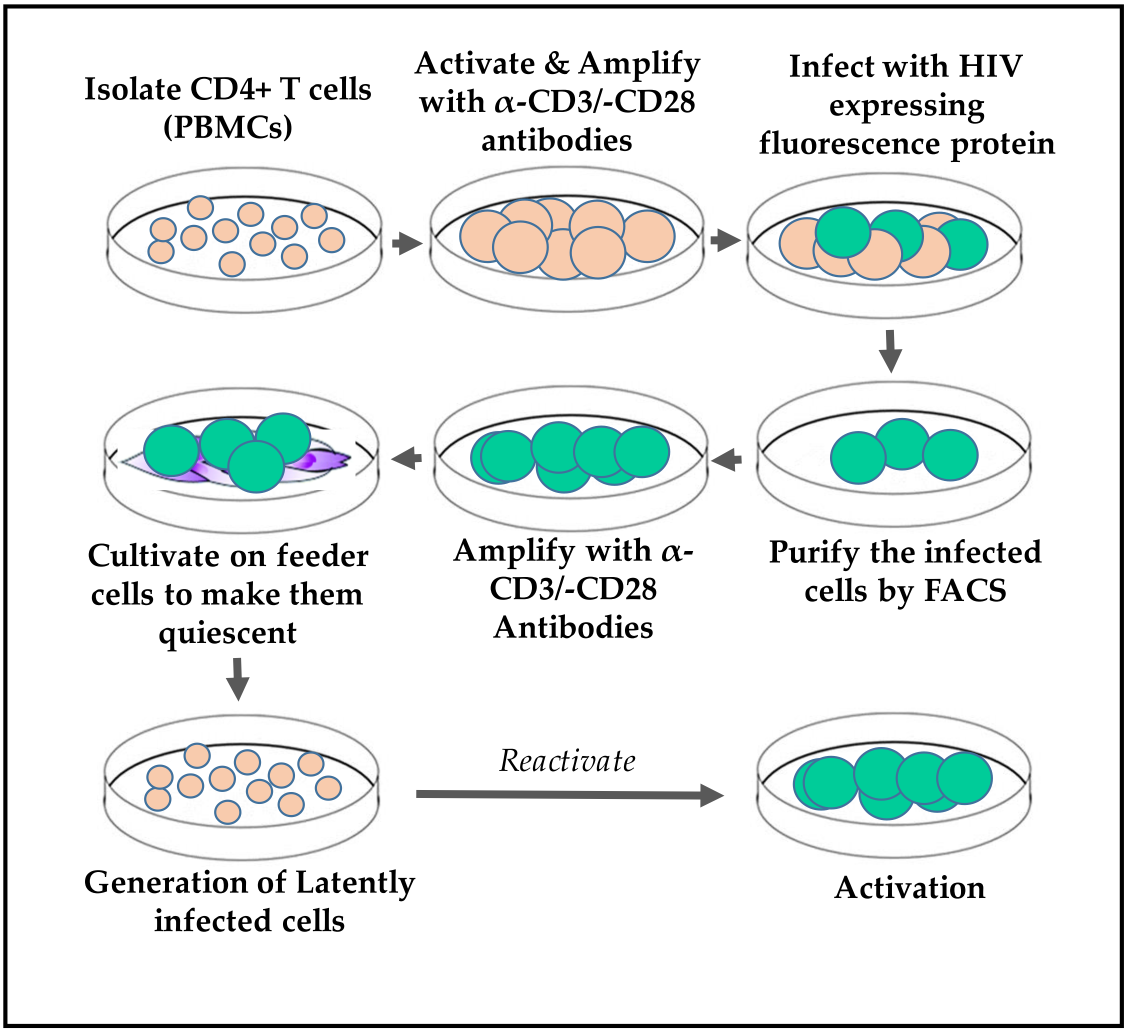

Supplementary Figure 1: Schematic representation of Tyagi-Sahu model, which was used to generate latently infected primary CD4+ T cells.

CBF-1 promotes the establishment and maintenance of HIV latency by recruiting Polycomb repressive complexes, PRC1 and PRC2, at HIV LTR

Supplementary Figure 2

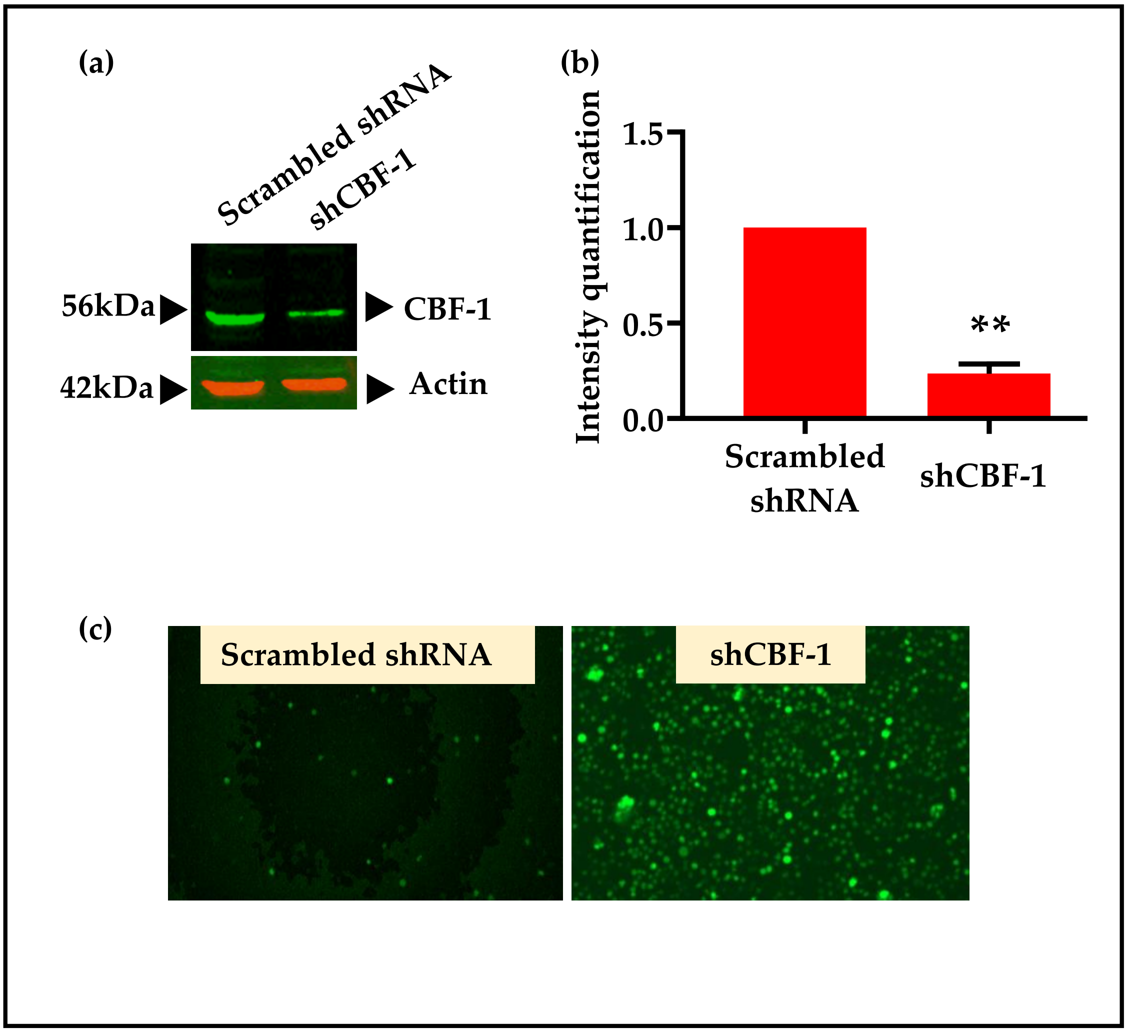

Supplementary Figure 2: CBF-1 knockdown disrupts the latency maintenance and leads to the proviral reactivation in latently HIV infected jurkat T cells (2D10 cells, a Jurkat cell with latently infected HIV). 2D10 cells were infected with lentiviral vectors expressing shRNAs either against CBF-1 or scrambled shRNA (a) nuclear lysate was then analyzed in western blot to check the level of knockdown (b) densitometry analysis normalized to actin and significant differences in protein expression were evaluated; $p < 0.01$ (**) against the controls. (c) cells were also observed under microscope for GFP expression after 48hrs to check the level of activation after shCBF-1. The microscopy image was taken at 10X resolution and the parameter used for images are same.

CBF-1 promotes the establishment and maintenance of HIV latency by recruiting Polycomb repressive complexes, PRC1 and PRC2, at HIV LTR

Supplementary Figure 3

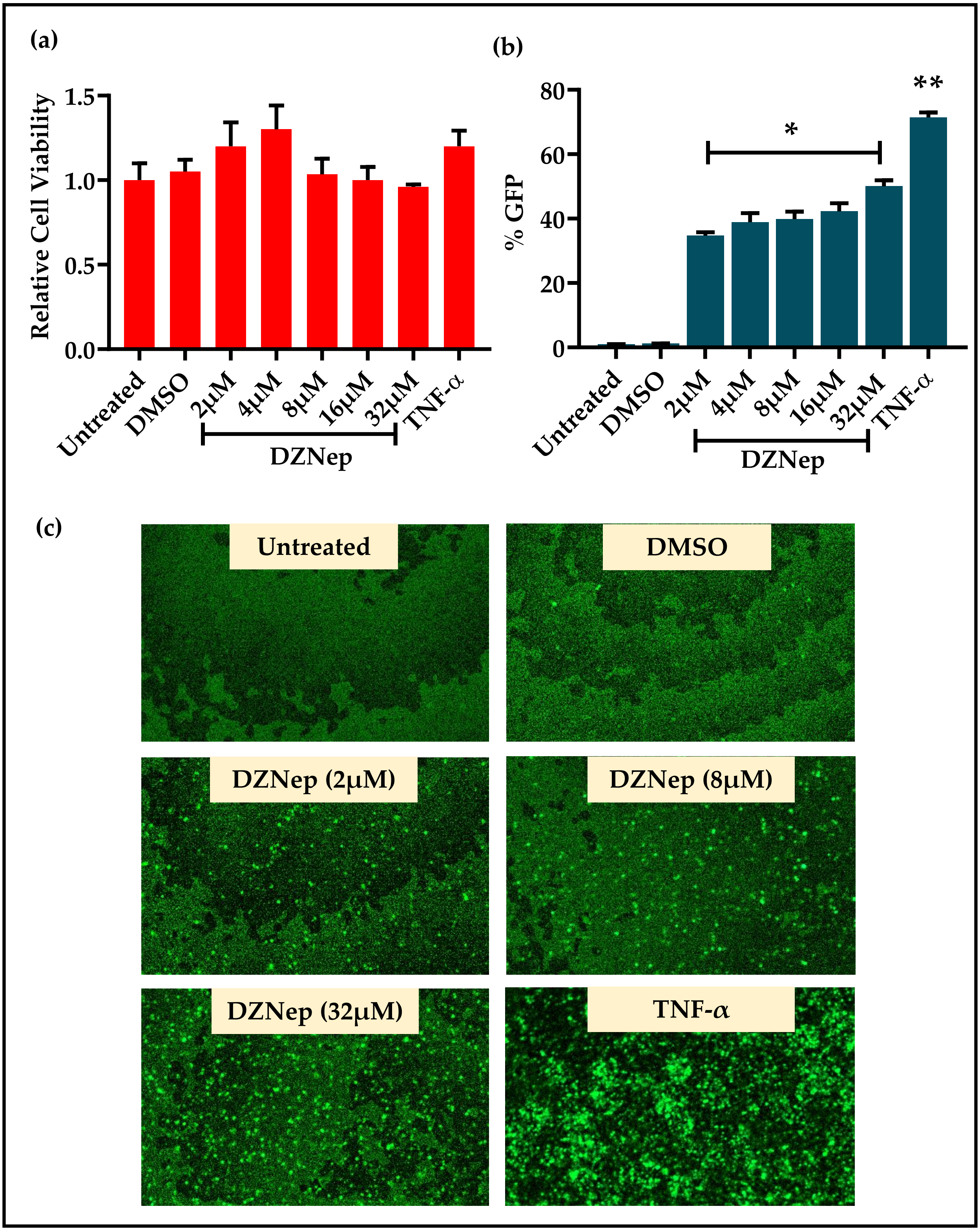

Supplementary Figure 3: The latently infected Jurkat T cells (2D10) were treated with DZNep (2μM-32μM), TNF-α (positive control) and DMSO (negative control). After 48 hrs., cells were assessed for cell viability (a), and GFP expression was evaluated via FACS (b) and fluorescent microscopy (c). Graphs represent the average and standard deviation from three independent and replicate samples. Statistical analysis was calculated with GraphPad Prism 5.0 (GraphPad Software, San Diego, CA, USA). The p value of statistical significance was set at either; $p < 0.05$ (*), 0.01 (**) or 0.001 (***).

CBF-1 promotes the establishment and maintenance of HIV latency by recruiting Polycomb repressive complexes, PRC1 and PRC2, at HIV LTR

Supplementary Figure 4

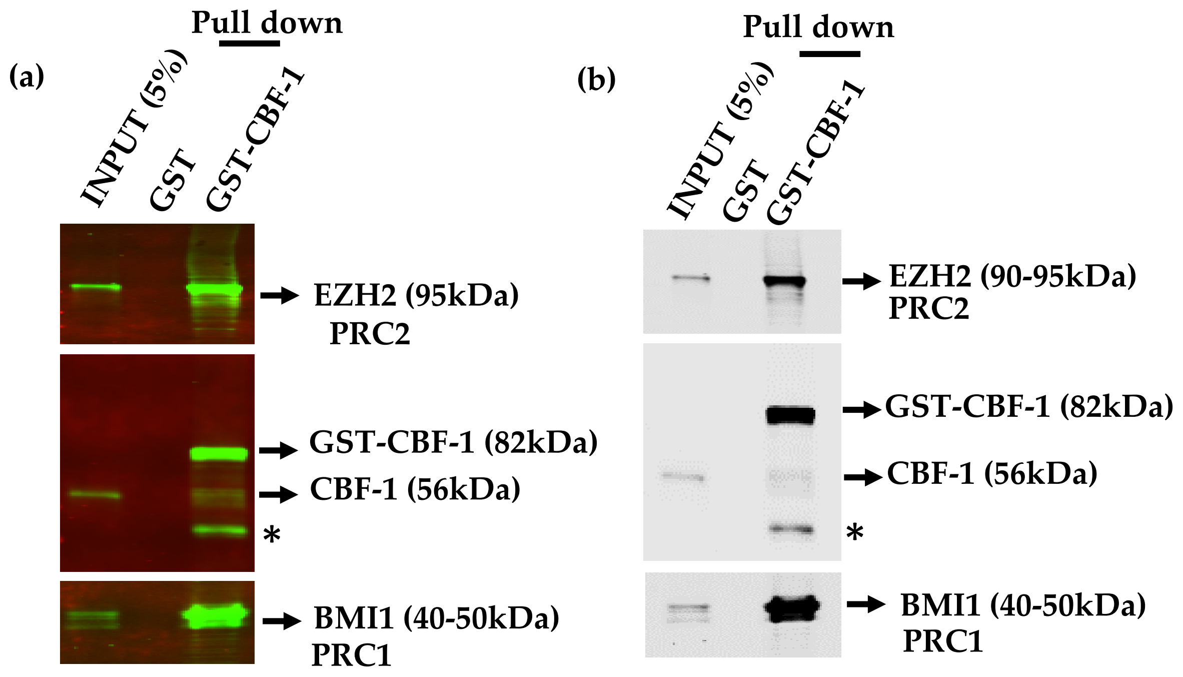

Supplementary Figure 4: CBF-1 interacts with BMI1 (PRC1) and EZH2 (PRC2) *in vitro*. (a & b) GST pull-down assay. Western blot analysis of CBF-1, BMI1 (PRC1) and EZH2 (PRC2) obtained from a GST pull-down assay using GST (lane 2) or GST-CBF-1 (lane 3). * denotes non specific. Five percent of the input was loaded (lane 1). (a) and (b) are same blot image but with green channel (left panels) and gray scale (right panels).
